# Exploration of the mechanism of luteolin against ischemic stroke based on network pharmacology, molecular docking and experimental verification

**DOI:** 10.1080/21655979.2021.2006966

**Published:** 2021-12-19

**Authors:** Rui Dong, Renxuan Huang, Xiaohua Shi, Zhongxin Xu, Jing Mang

**Affiliations:** a Department of Neurology, China-Japan Union Hospital of Jilin University; b Department of Neurosurgery, China-Japan Union Hospital of Jilin University

**Keywords:** Luteolin, ischemic stroke, network pharmacology, target prediction, enrichment analysis

## Abstract

Stroke is a leading cause of morbidity and mortality worldwide. As the most common type of stroke cases, treatment effectiveness is still limited despite intensive research. Recently, traditional Chinese medicine has attracted attention because of potential benefits for stroke treatment. Among these, luteolin, a natural plant flavonoid compound, offers neuroprotection following against ischemic stroke, although the specific mechanisms are unknown. Here we used network pharmacology, molecular docking, and experimental verification to explore the mechanisms whereby luteolin can benefit stroke recovery. The pharmacological and molecular properties of luteolin were obtained from Traditional Chinese Medicine Systems Pharmacology Database and Analysis Platform. The potential targets of luteolin and ischemic stroke were collected from interrogating public databases. Gene Ontology and Kyoto Encyclopedia of Genes and Genomes pathway analyses were performed by Funrich and Database for Annotation, Visualization and Integrated Discovery respectively, a luteolin-target-pathway network constructed using Cytoscape, Autodock vina was used for molecular docking simulation with Discovery Studio was used to visualize and analyze the docked conformations. Lastly, we employed an in vitro model of stroke injury to evaluate the effects of luteolin on cell survival and expression of the putative targets. From 95 candidate luteolin target genes, our analysis identified six core targets . KEGG analysis of the candidate targets identified that luteolin provides therapeutic effects on stroke through TNF signaling and other pathways. Our experimental analyses confirmed the conclusions analyzed above. In summary, the molecular and pharmacological mechanisms of luteolin against stroke are indicated in our study from a systematic perspective.

## 1. Introduction

Stroke is a leading cause of death worldwide [1–4] and its incidence continues to increase annually due to aging populations [5]. The most common type involves ischemic stroke which results from blood flow reduction to the brain parenchyma as well as thromboembolic occlusion of the cerebral artery [6–8]. The resulting cellular stress can trigger a number of pathogenic events, such as excitotoxicity, oxidative stress, and mitochondrial disturbances [9], which can affect post-stroke recovery and patient prognosis [10]. Focal cerebral ischemia also induces neuroinflammation where there is a time-dependent recruitment and activation of inflammatory cells resulting in neuronal cell death through the release of large amounts of inflammatory factors, This process particularly includes the resident innate immune cells known as microglia which can be activated and polarized to pro-inflammatory and anti-inflammatory phenotypes [4,8,11]. The current primary treatment options for ischemic stroke are thrombolytic therapy and thrombectomy [10,12]. However, the effectiveness of treatments is restricted by a narrow therapeutic window. Moreover, the injudicious timing of treatments may result in additional damage and exacerbate neurocognitive deficits [6,13]. Therefore, finding more effective treatment options for stroke is an urgent clinical need.

Chinese medicine, given as a single compound or a combination of different compounds, has been proven to be effective in the treatment of ischemic stroke during the acute and sub-acute phase. Indeed, a number of clinical trials have explored the efficacy of Chinese medicine with results showing these agents can complement traditional Western treatment strategies and benefit patient recoveries after stroke [14,15]. Positive effects recorded include attenuating cellular oxidative stress, alleviating excitotoxicity, maintaining the integrity of the blood-brain barrier (BBB) together with the functional integrity of neurovascular units [16,17]. Among the natural agents currently evaluated is luteolin (3’,4’,5,7-tetrahydroxyflavone), a flavonoid compound that is widely found in different plant species [18]. It has been shown that peripheral administration of luteolin can freely penetrate the BBB and provide neuroprotective effects against brain damage [19]. For example, luteolin was shown to act against ischemia and reperfusion injury, acting to reduce the size of the infarct area in an experimental model [20]. Moreover, a clinical trial evaluating recovery after cerebral ischemia reported that treating stroke patients with luteolin for 60 days resulted in improvements in different functional scores including the Canadian Neurological Scale (CNS), Mini-Mental State Examination (MMSE), and Ashworth Scale [13]. Different investigations have reported a wide range of beneficial effects of luteolin which may explain its neuroprotective actions including anti-inflammation, anti-allergic, anti-viral, antioxidant and anti-proliferative activities [20–22]. However, the specific mechanisms involved in the neuroprotective effects of luteolin against ischemic stroke have not been currently defined.

Network pharmacology is a systematic approach to discover relationships between diseases, targets and drugs, helping to understand the pharmacology of drugs and their impact on biological networks [23]. To explore the hypothesis that luteolin has a neuroprotective effect against brain damage, our study aimed to uncover the neuroprotective mechanisms whereby luteolin protects against ischemic stroke using network pharmacology analysis. This approach provides data outputs including PPI networks, GO analysis, KEGG pathway enrichment analysis and molecular docking. Based on these analyses, we then undertook experimental verification using an in vitro model that simulates ischemic stroke. Our integrated findings offer new mechanistic insights into the underlying genes and pathways targeted by luteolin, providing practical and theoretical knowledge to advance treatment options for patient recovery after ischemic stroke.

## 2. Materials and methods

### 2.1 ADME-related properties of luteolin

We determined the ADME-related properties of luteolin. The traditional Chinese medicine systems pharmacology database and analysis platform TCMSP (https://tcmspw.com/tsps) is a unique systemic pharmacology platform used to capture the relationships between herbal medicines, targets and diseases, which provides pharmacokinetic characteristics of natural mixtures, such as oral bioavailability (OB), drug-likeness (DL), intestinal epithelial permeability (Caco-2), water-solubility, etc [24]. In this study, we accessed the TCMSP to determine the ADME-related properties of luteolin by searching for the chemical name ‘luteolin’ to collect available information about its pharmacokinetic and other relevant properties.

### 2.2 Collection of targets of ischemic stroke and luteolin associated with ischemic stroke

Then we collected the targets of ischemic stroke and luteolin associated with ischemic stroke via different databases in order to prepare for the subsequent analysis. Ischemic-related targets were determined by searching public databases, including Online Mendelian Inheritance in Man (OMIM, https://omim.org/), a comprehensive compendium of human genes and genetic phenotypes, which focuses on the relationship between phenotype and genotype; DisGeNET (v7.0) (http://www.disgenet.org/), a platform containing publicly available collections of genes associated to human diseases that used to validate computationally predicted disease genes; DrugBank (https://go.drugbank.com/), a comprehensive database to combine detailed drug data with comprehensive drug target information [25]; and Therapeutic Target Database 2020 (TTD) (http://db.idrblab.net/ttd/), a database to provide information about the targeted disease, pathway information [26]. Finally, the gene symbols that correspond to the targets were derived from UniProt (http://www.uniprot.org/), a globally recognized knowledgebase used to access protein sequence and annotation data [27]. In parallel, we downloaded the SDF files for luteolin from PubChem, (https://pubchem.ncbi.nlm.nih.gov/), an authoritative web-based resource providing chemical information. The targets of luteolin were gathered from 3 public databases: PharmMapper (http://www.lilab-ecust.cn/pharmmapper/submitfile.html) used to identify potential target candidates of small molecules using a pharmacophore mapping approach [28], SwissTar getPrediction (www.swisstargetprediction.ch) used to perform ligand-based target prediction for bioactive small molecules [29], and Comparative Toxicogenomics Database (http://ctdbase.org/) used to curate and infer gene-disease associations [30]. Finally, we calculated the intersections between the targets of luteolin and ischemic stroke and plotted Draw Venn Diagram (http://bioinformatics.psb.ugent.be/webtools/Venn/) representing the overlapping genes. The potential target genes were entered into the STRING database(https://string-db.org/) which incorporates > 52 million proteins from > 1100 species, to derive the associated protein-protein interactions [31].

### 2.3 Construction of PPI networks and analysis of pivotal genes

To study the relationship between luteolin, targets and diseases, we used the network visualization software Cytoscape software (3.8.0) to construct and visualize PPI networks with a medium confidence level (0.4) [32]. Cytohubba and MCODE plug-ins were used to screen for hub genes and modules among the target genes, respectively. The parameters of CytoHubba were set as core genes = top 10 nodes ranked by degree, maximal clique centrality (MCC) and maximum neighborhood component (MNC). MCODE parameters were set to default.

### 2.4 GO analysis and KEGG pathway enrichment analysis

GO enrichment and KEGG pathway enrichment analysis were used to explore the potential functions of luteolin against complications of ischemic stroke. The targets of luteolin for treatment of ischemic stroke were calculated by FunRich software for GO analysis to derive statistically significant (P value<0.05) enrichments [33].

KEGG pathway analysis can give functional meaning to genes at the molecular level, and provide a systematic and comprehensive analysis of a defined list of target genes that are significantly enriched in defined biological pathways [34]. All luteolin targets related to the treatment of ischemic stroke were submitted to the DAVID Bioinformatics Resources 6.8 database (https://david.ncifcrf.gov/), which was widely used for gene function annotation and enrichment, to obtain the KEGG pathway analysis results [35,36]. Additionally, the bioinformatics platform (http://www.bioinformatics.com.cn/) was used to draw the bubble chart of KEGG enrichment analysis.

### 2.5 Molecular docking simulation

Molecular docking techniques between small molecules and relevant targets can, to a certain degree, predict binding mechanisms and activity between active ingredients and putative target proteins [37]. Toward the prediction of interactions between luteolin and target proteins, the luteolin structure from the PubChem database(https://pubchem.ncbi.nlm.nih.gov/) was converted from the SDF to the mol2 format using Chem 3D software. PDB format structures of the target proteins interleukins-1β(IL1B) (PDB ID: 5R85), JUN (PDB ID: 2G01), Mitogen-activated protein kinase 3 (MAPK3) (PDB ID: 6GES), Matrix metalloproteinase 9 (MMP9) (PDB ID: 4JIJ), Prostaglandin H synthase 2 (PTGS2) (PDB ID:5F19) and tumor necrosis factor (TNF) (PDB ID: 2AZ5) were downloaded from the RCSB (https: //www.rcsb.org/), a database which is established as the digital data resource in all of biology and medicine and provides access to 3D structure data for large biological molecules. The solvent molecules and ligands were removed by Pymol software, performed to pre-dock small molecule components and proteins. After using AutoDock Tools 1.5.6, a software for molecule docking based on the semi-flexible principle, to add hydrogen, calculate charges, assign atomic types and carry out other operations, the processed structures were saved as PDBQT format. Finally, Autodock vina 1.1.2 was run for molecular docking simulation to analyze the binding properties of the luteolin ligand to the target proteins, and Discovery Studio 2020 was used to visualize and analyze the docked conformations.

### 2.6 Cell culture *and OGD treatment*

PC-12 cells (Cell Bank of the Chinese Academy of Sciences) were employed as an in vitro model to simulate ischemic stroke using oxygen and glucose deprivation (OGD) treatment. Cells were maintained in Dulbecco’s Modified Eagle Medium (DMEM, Gibco, USA) containing 10% FBS(Invitrogen, USA) and 1% antibiotics (penicillin, 100 IU/ml; streptomycin, 100 μg/ml, Gibco, USA) at 37°C in 5% CO2 atmospheric conditions. To verify the neuroprotective effects of luteolin, PC-12 cells were pretreated with gradually increasing concentrations of luteolin(No. 491–70-3, MedChemExpress, China) (5 μM, 10 μM and 20 μM) for 24 hours prior to an 6-hour OGD treatment [38,39], consisting of refreshing the culture medium with glucose-free DMEM (Gibco, USA) before placing the cells in an anaerobic chamber (95% N2 and 5% CO2) for 6 hours [40]. Control group cells were cultured with complete medium and under normoxic conditions.

### 2.7 Cell viability assays

CCK-8 assays were used to evaluate cell viability according to the manufacturer’s instructions. Briefly, cells were seeded in 96-well plates at a density of 8000 cells per well. After treatment with the indicated combinations of luteolin and OGD, 10 μl CCK-8 reagent(Biosharp, Anhui, China) was added to each well and the cells were further incubated for 2 h at 37°C. Finally, the absorbance was read at 450 nm using Microplate Reader (Bio-Rad, USA) and cell viability was expressed as percentages relative to the control treatment.

2.8 qRT-PCR assays

The mRNA expression levels of MMP9, MAPK3, PTGS2, JUN, IL1B and TNF were determined using quantitative reverse-transcription polymerase chain reaction (qRT-PCR) analysis. Briefly, total cellular RNA was extracted using the Trizol reagent (TIANGEN, China) and reverse transcribed to cDNA using reverse transcription kit (No.FSQ-101, TOYOBO, Shanghai, China) according to the manufacturer’s instructions under the following conditions: 65°C (5 min), 37°C (15 min), and a final step of 98°C (5 min). qRT-PCR reactions were performed with 2X SYBR green qPCR master mix (B21023, bimake, USA) on a Bio-rad CFX-96 real-time PCR system(Bio-rad, USA) [41]. The specific primers of MMP9, MAPK3, PTGS2, JUN, IL1B and TNF (Sangon Biotech, Shanghai, China) for qRT-PCR are listed below (5’-3’):

TNFα: forward(TCAAGAGCCCTTGCCCTAAG) and reverse (TGGAAGACTCCTCCCAGGTA)

MMP9: forward(CCTGGAACTCACACAACGTC) and reverse (TGCAGGAGGTCATAGGTCAC)

JUN: forward(GAGTCTCAGGAGCGGATCAA) and reverse (CTGTTCCCTGAGCATGTTGG)

MAPK3: forward(AAGCGCATCACAGTAGAGGA) and reverse (TCAGCCACTGGTTCATCTGT)

IL1B: forward(GGGATGATGACGACCTGCTA) and reverse (TGTCGTTGCTTGTCTCTCCT)

PTGS2: forward(TCCAAACCAGCAGGCTCATA) and reverse (ATTCAGAGGCAATGCGGTTC)

### 2.9 Western blotting

The influence of luteolin on the expressions of MMP9, MAPK3, PTGS2, JUN, IL1B and TNF in protein level was assessed by Western blot. PC-12 cells were lysed before measuring the protein concentrations of the cell lysates using the BCA protein assay kit (Beyotime, China). Thereafter, 20 ug of protein/sample was separated using 12% SDS-PAGE gels (Yeasen, China) before electrophoretic transferred to the PVDF membranes. The membranes were then blocked with 5% skim milk (Absin, Shanghai, China) for 2 h at room temperature and followed by incubation overnight at 4°C with primary antibodies directed against MMP9(sc-393,859, Santa, USA), MAPK3(sc-271,269, Santa, USA), PTGS2(sc-376,861, Santa, USA), JUN (#9165, CST, USA), IL1B(sc-12,742, Santa, USA) and TNF (#11,948, CST, USA). After washing with TBST for 30 minutes at room temperature, we incubated the immune blots with secondary antibodies(#7074, CST, USA) for 2 h. Finally, proteins were visualized by Yeasen super ECL detection reagent Kit (Yeasen, China) using Syngene Bio Imaging instrument (Synoptics, Cambridge) and were quantified by Image J [42].

### 2.10 Statistical analysis

All values are expressed as mean ± SD with the student’s *t*-test used to perform statistical comparisons between groups. P < 0.05 was considered to indicate a statistically significant difference.

3.Results

In order to verify the neuroprotective effects of luteolin against ischemic stroke and to explore the mechanisms of neuroprotection, we first employed network pharmacology analysis. This was followed by experimental verification methods, including PPI networks, GO analysis, KEGG pathway enrichment analysis, molecular docking, and in vitro culture models of ischemic stroke assessed by cell survival assays, qRT-PCR and Western blotting.

### 3.1 ADME-related characteristics of luteolin

ADME parameters represent important steps in the drug discovery process. We obtained the

ADME characteristics of luteolin by interrogating the TCMSP database (Table 1), including DL, OB, Caco-2, BBB and ‘Lipinski’s Rule of Five’ (MW, AlogP, TPSA, Hdon and Hacc).

**Table 1.** Pharmacological properties of luteolin

Name	MW	AlogP	Hdon	Hacc	OB (%)	Caco-2	BBB	DL	TPSA
Luteolin	286.25	2.07	4	6	36.16	0.19	−0.84	0.25	111.13

### 3.2 PPI Networks and cluster analysis of stroke disease targets

**Figure 1.**
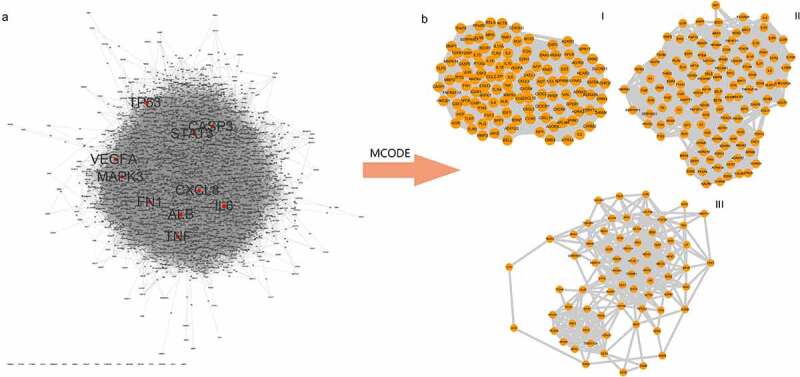
PPI network and Analysis of disease target clusters. (a) PPI network of stroke targets. The middle red nodes represent the top 10 genes with the highest degree values. (b) The three major clusters from the PPI network for stroke targets

This list was about 1200 stroke-related genes, among which 1159 genes were from the DisGeNET database, 77 from Drugbank, 12 from TTD, and 180 from OMIM (Figure 4b, Table S1-S5). We used the STRING online database service platform to derive functional protein association networks associated with stroke target proteins as shown in Figure 1. The middle ten nodes corresponded to the 10 genes with the highest degree values that are most important in the development and progression of stroke including TP53, TNF, CASP3, STAT3, MAPK3, CXCL3, FN1, IL6, VEGFA and ALB (Figure 1a). The PPI network of stroke targets was then clustered using the MCODE plugin in Cytoscape. We selected the top 3 clusters based on their scores (Figure 1b) and performed GO functional enrichment and KEGG pathway analysis on the targets covered by these 3 clusters.

### 3.3 GO analysis of stroke target genes

**Figure 2.**
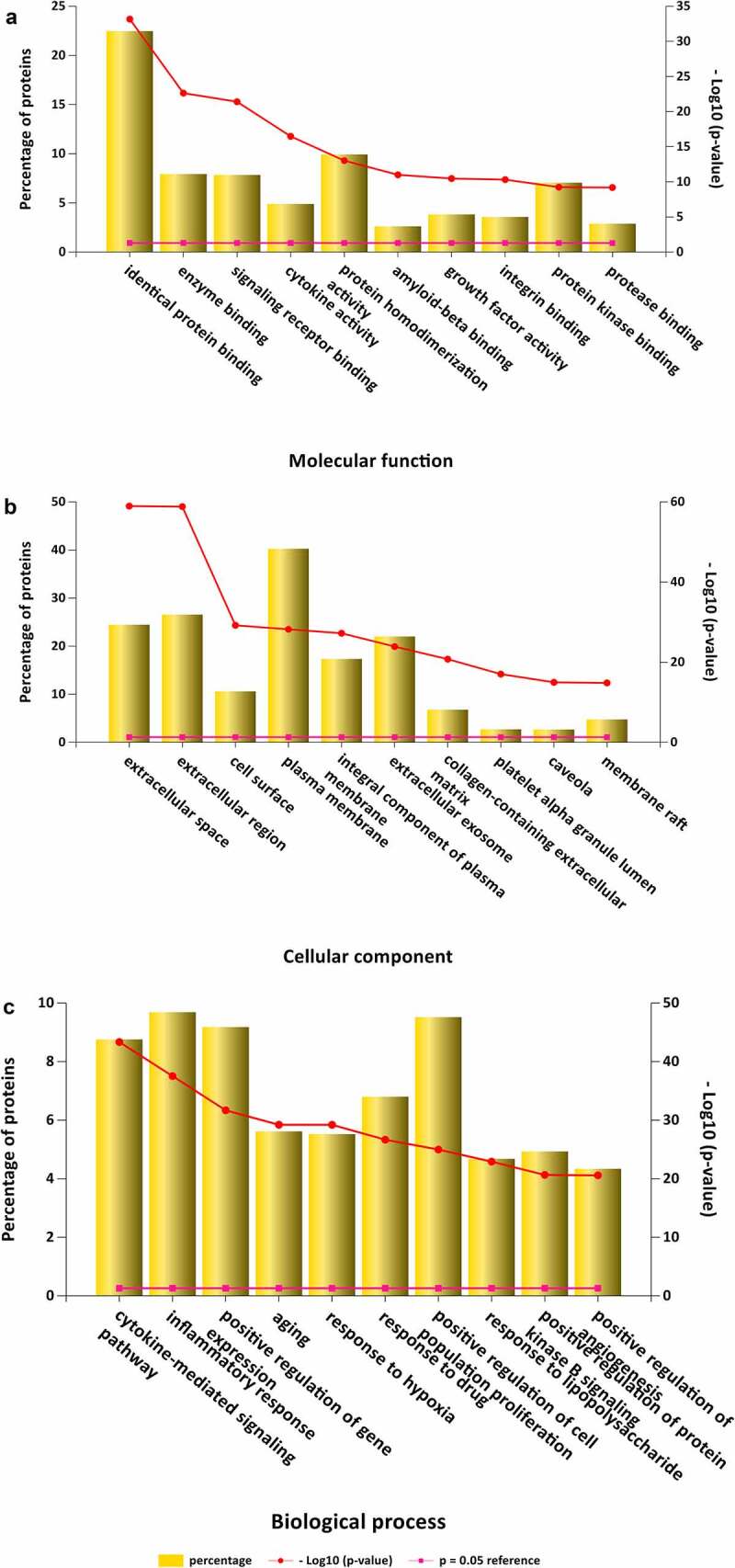
GO analysis for target genes of stroke. (a) 10 targets of stroke enriched of MF items; (b) 10 targets of stroke enriched of CC items; (c) 10 targets of stroke enriched of BP items. (P value < 0.05 for GO enrichment were considered statistically significant)

GO is a comprehensive resource that classifies gene functions into 3 main terms: biological processes (BPs), cellular components (CCs), and molecular functions (MFs) [34].The results revealed the top 10 MF enrichment items included identical protein binding, enzyme binding, signaling receptor binding, cytokine activity, protein homodimerization activity, amyloid-beta binding, growth factor activity, integrin binding, protein kinase binding and protease binding (Figure 2a). The top 10 CC enrichments included extracellular space, extracellular region, cell surface, plasma membrane, integral component of the plasma membrane, extracellular exosome, collagen-containing extracellular matrix, platelet alpha granule lumen, caveola and membrane rafts (Figure 2b). Lastly, the top 10 BP enrichment terms obtained were cytokine-mediated signaling pathway, inflammatory response, positive regulation of gene expression, aging, response to hypoxia, positive proliferation response to drug, positive regulation of cell, kinase B signaling response to lipopolysaccharide, angiogenesis positive regulation of protein and positive regulation of angiogenesis (Figure 2c).

### 3.4 KEGG pathway enrichment analysis for stroke target genes

**Figure 3.**
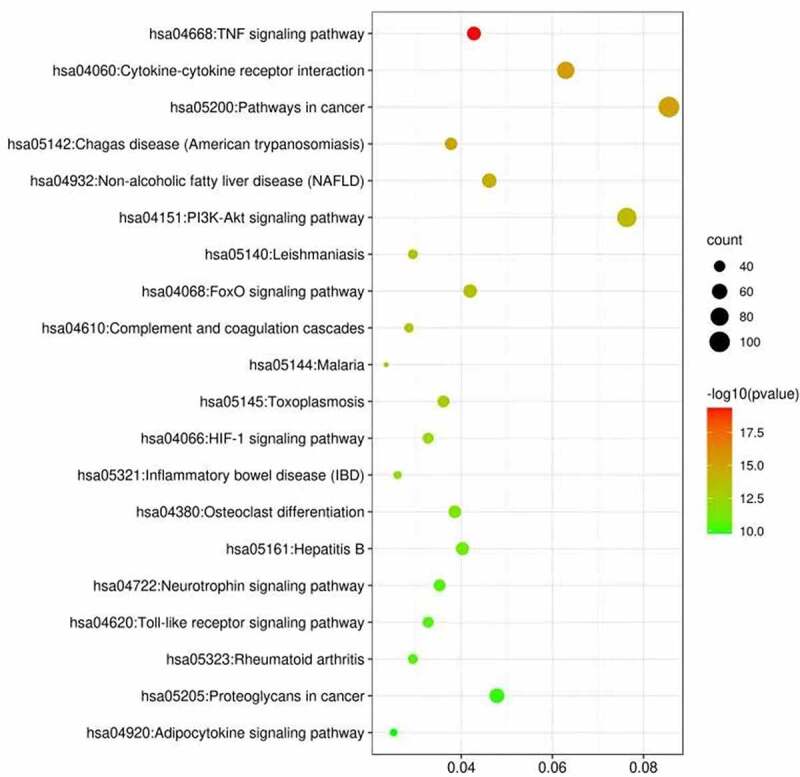
KEGG analysis of stroke target genes

Using DAVID 6.8, we performed KEGG enrichment analysis for the targets related to the treatment of ischemic stroke. This analysis retrieved a total of 38 significantly associated signaling pathways (p < 0.05; Table S6). Of these, the top 20 enriched pathways in stroke are illustrated in Figure 3 where notably the ‘TNF signaling pathway’ contained the largest number of targets and had the lowest p-value (4.34E-20).

**Figure 4.**
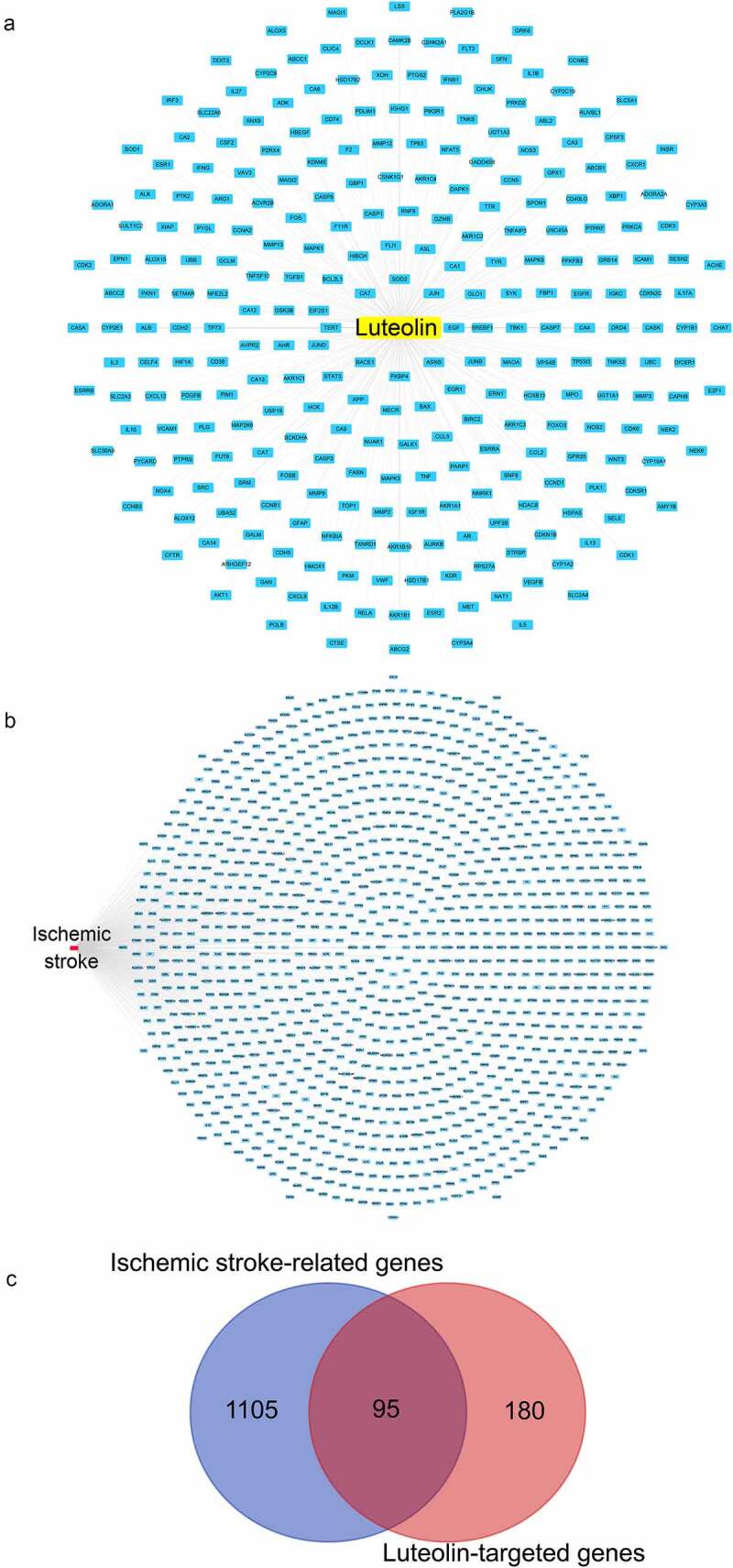
Luteolin- and stroke-related target genes. (a) Harvesting 275 luteolin-targeted genes through CTD, PharmMapper and SwissTargetPrediction databases, luteolin and luteolin-targeted genes are respectively represented as a yellow round rectangle and blue round rectangles. (b) Harvesting 1200 stroke- associated genes through OMIM, TTD, DisGeNET and Genecards databases, stroke and stroke-associated genes are respectively represented as a red rounded rectangle and blue rounded rectangles. (c) Analysis of 95 genes between 275 stroke-associated genes and 1200 stroke-associated genes via Draw Venn Diagram

### 3.5 Identification of the target genes related to stroke and luteolin

We compiled a total of 275 luteolin target genes from different sources including 111 from the Swiss target prediction, 124 from the CTD database, and 71 from Pharm-Mapper (Figure 4a, Table S7). Intersection analysis provided 95 genes in common between luteolin and stroke (Figure 4c) that were utilized to further study mechanisms whereby luteolin promotes stroke recovery.

### 3.6 PPI network of luteolin stroke target genes

**Figure 5.**
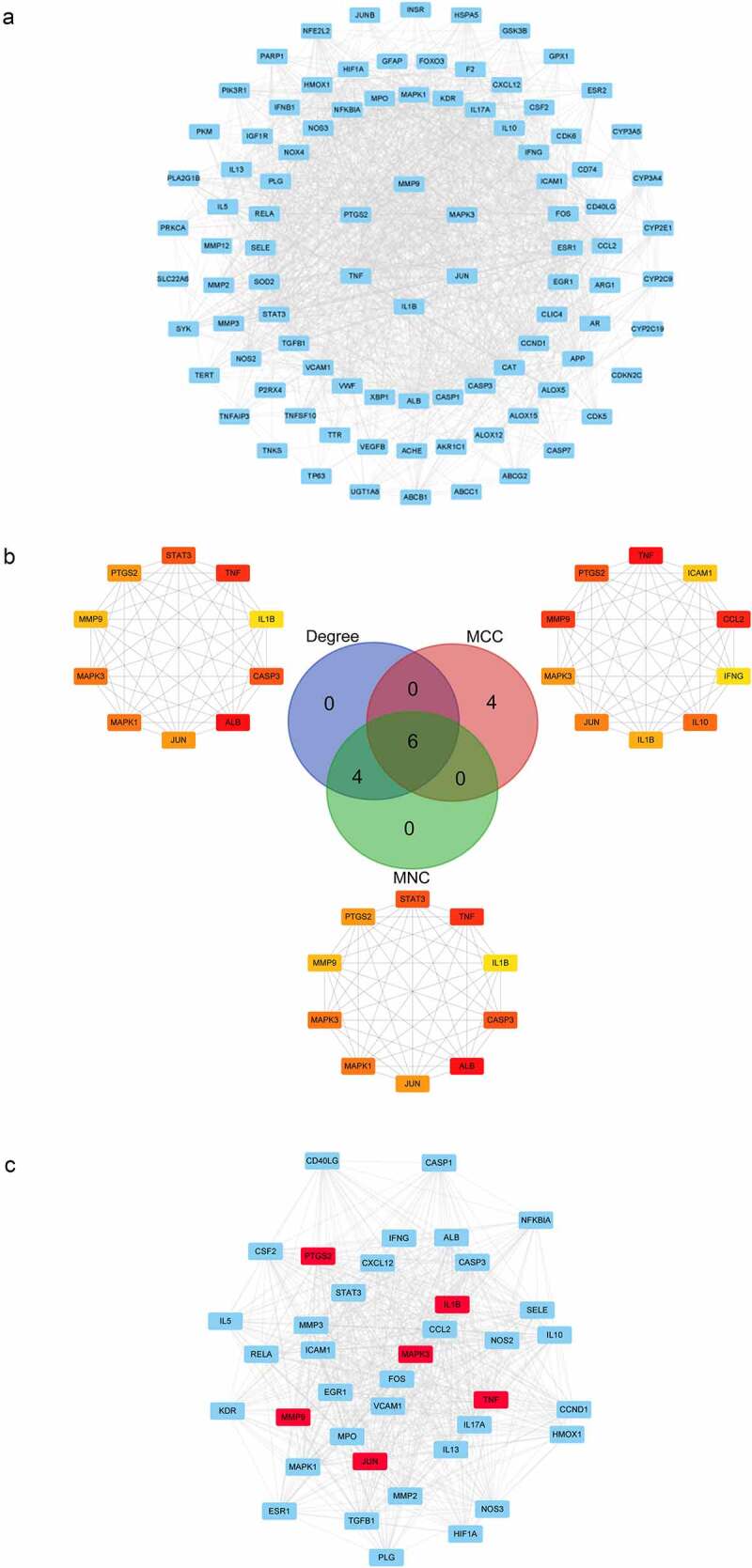
The PPI network luteolin anti-stroke and core genes were analyzed using CytoHubba and MCODE. (a) PPI network of luteolin anti-stroke. 95 blue round rectangles represent luteolin anti-stroke targets and 1397 edges represent target-to-target interactions. (b) PPI networks were analyzed using CytoHubba, sorted by degree, MNC and MCC, and then using the Draw Venn Diagram to obtain overlapping genes representing core genes. (c) Analysis of the most important modules using MCODE, with red round rectangles representing the overlapping genes (core genes) contained in (B)

Targets and interactions between targets are represented respectively by nodes and edges in a PPI network. Interrogation of the STRING database with the 95 luteolin stroke target genes produced a PPI network with 95 nodes and 1397 edges. The initial PPI network was built by Cytoscape 3.8.0 where the circles represent targets of luteolin against stroke, and the edges represent interactions between targets (Figure 5a). Analysis using CytoHubba yielded six core genes, including MMP9 and MAPK3 (Figure 5b). The MCODE results detected 39 modules (densely connected regions) scored as 32.368 within the ISA network which included 615 edges (Figure 5c). Of these, it is notable that the most important modules consisted of six core genes.

### 3.7 Luteolin-target-pathway network

**Figure 6.**
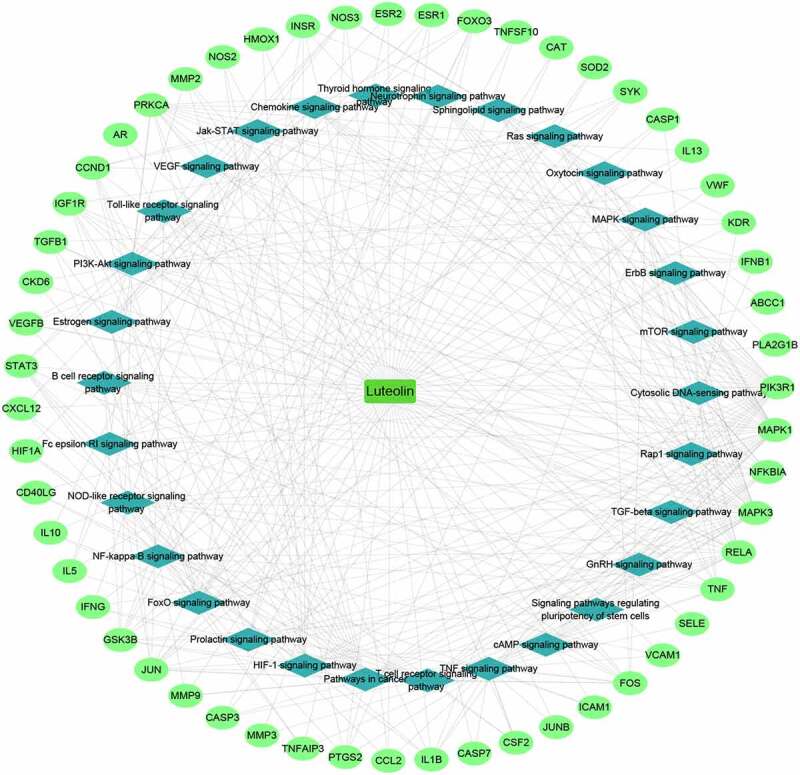
Luteolin-target-pathway network

The luteolin-target-pathway network was then visualized using Cytoscape 3.8.0 to derive the key biological mechanisms (Figure 6). This analysis revealed 86 nodes and 350 edges, uncovering the key signaling pathways underlying the activity of luteolin against stroke which included the TNF signaling pathway, PI3K-Akt signaling pathway, HIF-1 signaling pathway, TGF-beta signaling pathway, NF-kappa B signaling pathway, FoxO signaling pathway, and VEGF signaling pathway.

### 3.8 GO analysis of luteolin stroke target genes

**Figure 7.**
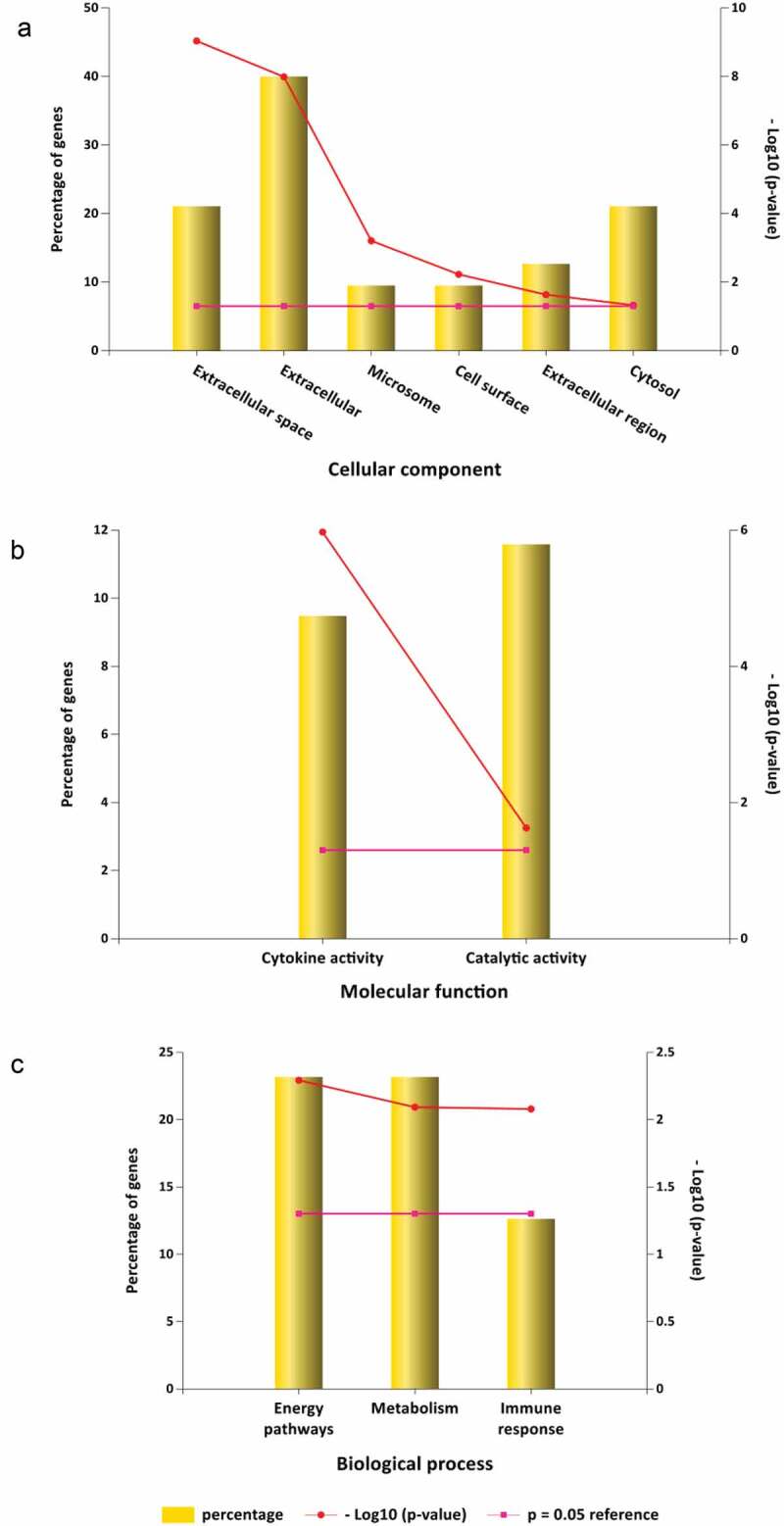
GO analysis for luteolin anti-inflammatory targets. (a) 6 luteolin anti-inflammatory targets enriched of CC items; (b) 2 luteolin anti-inflammatory targets enriched of MF items; (c) 3 luteolin anti-inflammatory targets enriched of BP items. (P value < 0.05 for GO enrichment were considered statistically significant)

The input of the 95 potential luteolin stroke target genes derived 6 CC enrichment items including extracellular space, extracellular, microsome, cell surface, extracellular region and cytosol (Figure 7a). MF enrichments provided two functions involving cytokine activity and catalytic activity (Figure 7b) and 3 BP enrichment terms involving energy pathways, metabolism and immune response (Figure 7c).

### 3.9 KEGG pathway enrichment analysis of luteolin stroke target genes

**Figure 8.**
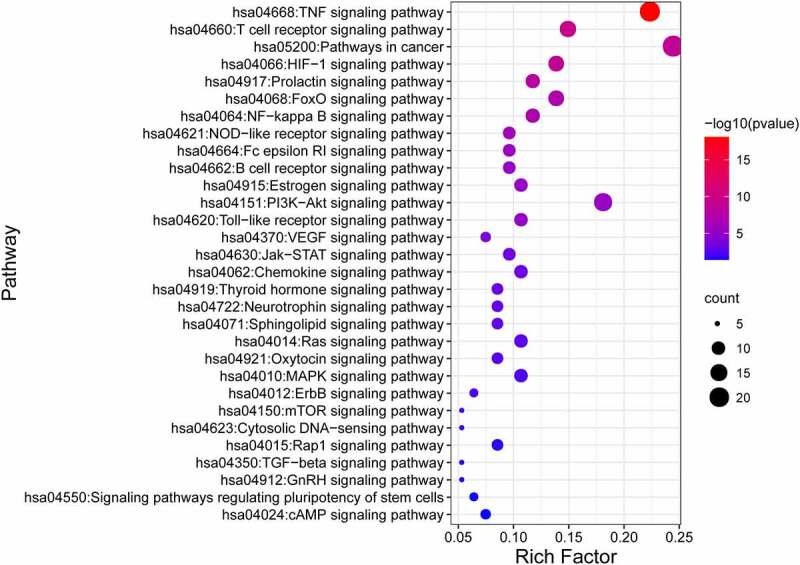
KEGG pathway analysis for target genes of luteolin against stroke

KEGG analysis against the 95 common gene targets between luteolin and stroke showed enrichments in 28 signaling pathways (p < 0.05; Table S8) suggesting these pathways are likely targeted by luteolin to perform a therapeutic role in stroke. The top 30 significantly enriched KEGG pathways are shown in Figure 8, of which the TNF signaling pathway comprised the maximum number of targets with the most significant P-value (8.34E-19).

**Figure 9.**
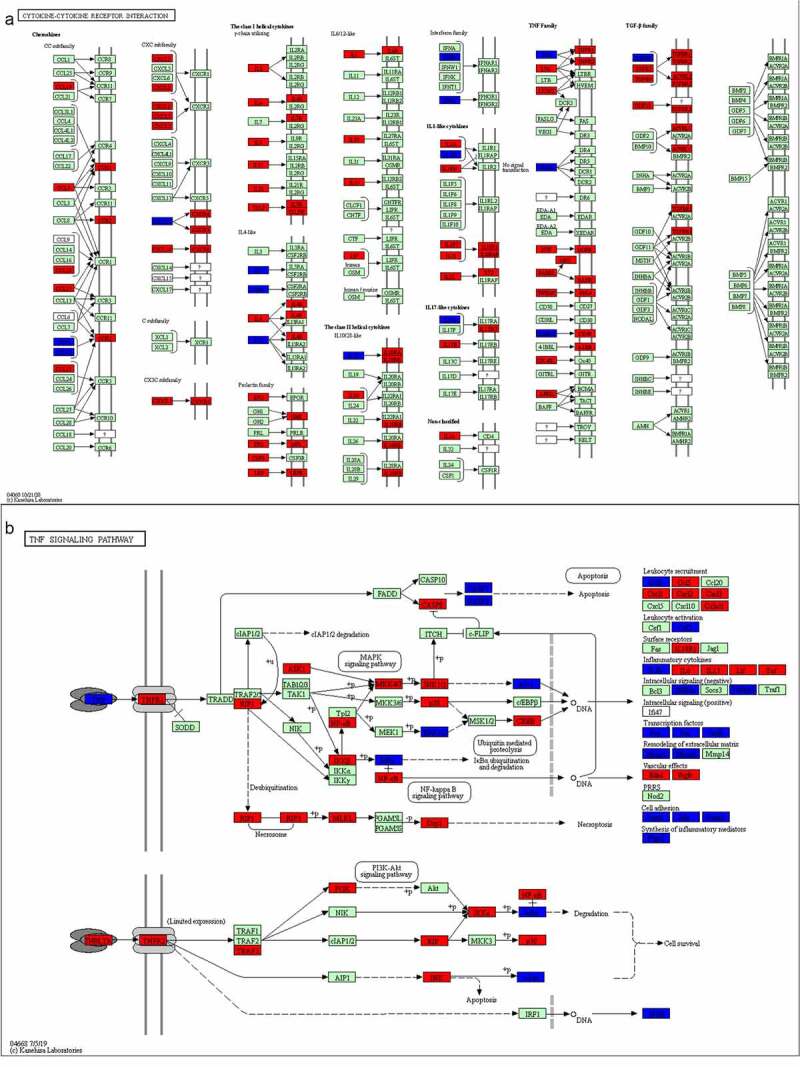
A comprehensive pathway map of luteolin against stroke

### 3.10 Pathway map of stroke and luteolin related targets

Based on the luteolin-targeting-pathway network, we constructed the integrated pathway map by integrating the stroke pathway map and signaling pathways obtained from the assessment of the targeted pathway network to visualize the complex mechanism of luteolin anti-stroke (Figure 9). As shown in Figure 9a, the red rectangles represent targets of stroke, while luteolin targets are represented as blue rectangles. The cytokine-cytokine receptor interaction consisted of chemokines, class I helical cytokines, class II helical cytokines, IL-1-like cytokines, IL-17-like cytokines, and the TNF family and TGF-β family, of which TNF and IL1B were notably derived from the PPI network analysis incorporating luteolin and stroke as described above. Additionally, both the class I helical cytokines and the TNF family showed many stroke targets, proposing these as the most likely focus of stroke treatments.

In terms of key signaling pathways (Figure 9b), based on analysis of the luteolin-stroke PPI network, TNF was proposed to be one of the six pivotal genes targeted by luteolin in the treatment of stroke. Moreover, this result is consistent with the GO and the KEGG pathway enrichment analysis of the target genes of luteolin in stroke treatment. Together these findings suggest that TNF plays an important role in the therapeutic actions of luteolin when employed for the treatment of stroke.

### 3.11 *Confirmation about* molecular docking simulation of luteolin against stroke

**Figure 10.**
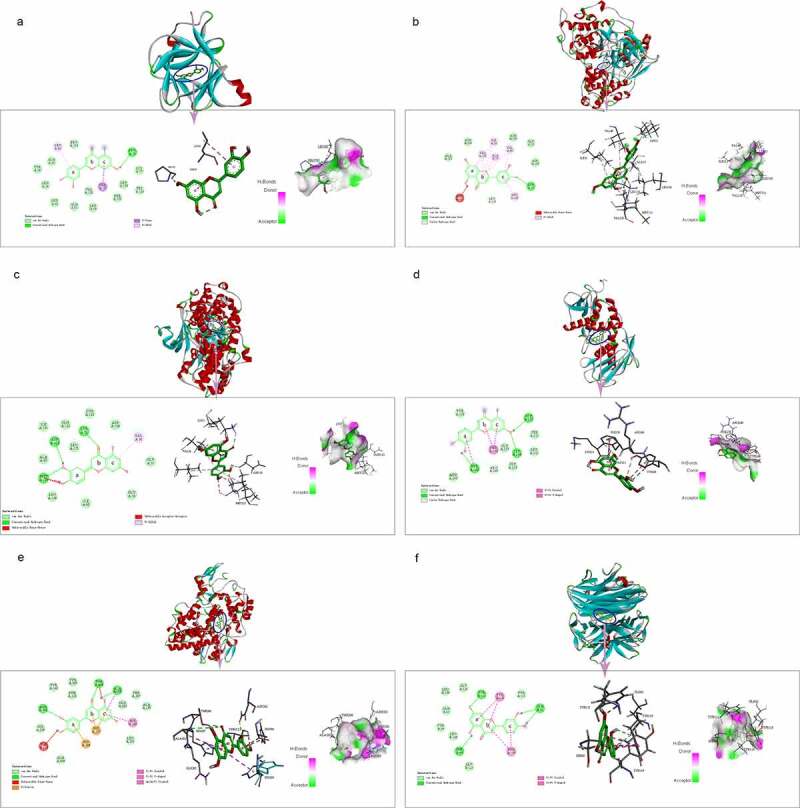
The molecular docking between luteolin and target proteins. (a). Molecular docking of IL1B (PDB ID: 5R85) with luteolin. (b). Molecular docking of JUN (PDB ID: 2G01) with luteolin. (c). Molecular docking of JUN protein MAPK3 (PDB ID: 6GES) with luteolin. (d) Molecular docking of MMP9 (PDB ID: 4JIJ) with luteolin. (e) Molecular docking of PTGS2 (PDB ID:5F19) with luteolin. (f). Molecular docking of TNF (PDB ID: 2AZ5) with luteolin

Based on the PPI network, six core target proteins were selected for molecular docking experiments against luteolin (Figure 10). The binding affinity of target proteins to luteolin was shown by comparison with the target protein’s reported ligands (Table 3). The results expressed as Docking Scores suggested that luteolin has similar, even more potent effects compared to prototypical ligands such as IL1B, JUN, MAPK3, MMP9, PTGS2 and TNF. The bonding interaction data are shown in Tables 2 and 3, respectively.

**Figure 11.**
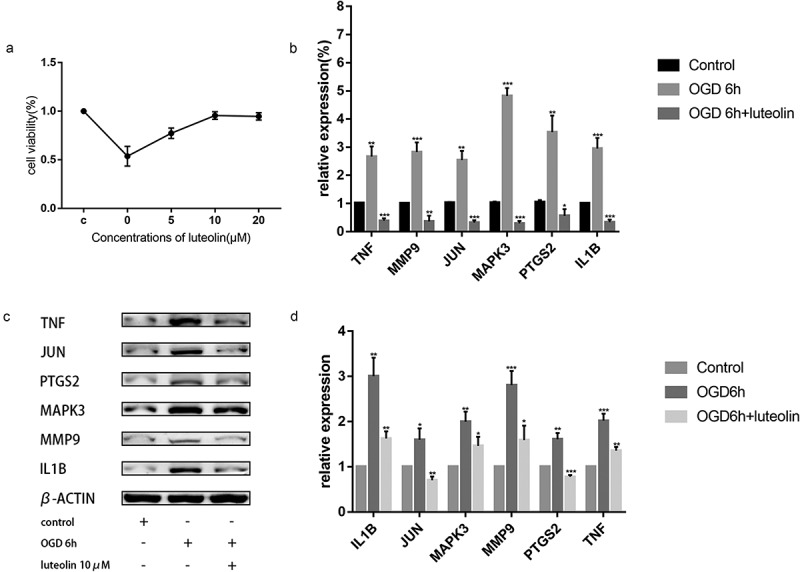
(a). Cell viability after OGD with different doses of **l**uteolin determined by CCK8. (b). Effects of luteolin on the mRNA levels of MMP9, MAPK3, PTGS2, JUN, IL1B and TNF of PC12 cells. *P < 0.05,**P < 0.01,***P < 0.001 vs. the control group. (c). The expression of MMP9, MAPK3, PTGS2, JUN, IL1B and TNF were examined by Western blotting. (d). Effects of luteolin on the expression levels of MMP9, MAPK3, PTGS2, JUN, IL1B and TNF of PC12 cells. *P < 0.05,**P < 0.01,***P < 0.001 vs. the control group

**Table 2.** 

Protein	PDB ID	Ligands	Affinity (kcal/mol)	original ligands	Affinity (kcal/mol)
IL1B	5R85	Luteolin	−7.5	S7A	−5.6
JUN	2G01	Luteolin	−8.9	73Q	−7.7
MAPK3	6GES	Luteolin	−8	6H3	−7.9
MMP9	4JIJ	Luteolin	−7.1	PEG	−2.8
PTGS2	5F19	Luteolin	−9.3	COH	−12.8
TNF	2AZ5	Luteolin	−7.9	307	−9.3

**Table 3.** 

Protein		A-ring	B-ring	C-ring
IL1B	Hydrophobic interaction	Leu80		
	π-sigma interaction			Thr79
	hydrogen bonding interaction			Pro78
JUN	hydrophobic interaction	Val158 and Ile32	Val158, Ile32, Ala53, Val40	Val40, Leu168 and Lys55
	hydrogen bonding interaction		Carbonyl B-ring with Leu110	Lys55
MAPK3	hydrophobic interaction			Val56
	hydrogen bonding interaction	Asp123 and Met125	Lys71	
MMP9	π-π ring stacking effect	Tyr248	Phe250	Phe250
	hydrogen bonding interaction	Tyr248 and Arg249		Gly215 and Lys214
PTGS2	π-cationic interaction	His386	His207	His207
	hydrogen bonding interaction	Asn382	Thr206	Ala202
	π-π ring stacking effect			His388
	amide-π stacking effect			Ala202
TNF	π-π ring stacking effect	Tyr119	Tyr119	Tyr119
	hydrogen bonding interaction	Tyr151 and Ser60		Gln61

### 3.12 L*uteolin* prevents OGD-induced cell death in PC-12 cells by inhibiting target gene expression

Ischemic conditions in the brain can be modeled in vitro through OGD. We assessed the effects of luteolin on OGD-induced injury in PC-12 cells, a cell line that closely resembles neurons. We first pretreated PC-12 cells with 5, 10 or 20 μM luteolin for 24 hours prior to conducting OGD treatment for 6 hours (Figure 11A). Assessment of cell viability using CCK-8 assays indicated that luteolin maintained the viability of PC-12 cells following OGD in a concentration-dependent manner with maximal effects achieved at 10 μM luteolin. These findings indicated that the neuroprotective effects of luteolin in stroke could be readily modeled in vitro. Furthermore, the experiments also established an optimal treatment dose of luteolin for use in subsequent experiments.

Next, we sought to establish whether luteolin pretreatment could decrease the expression levels of MMP9, MAPK3, PTGS2, JUN, IL1B and TNF after OGD-induced injury. Measurement of their mRNA and protein expressions levels by qRT-PCR and Western blotting, respectively, showed that OGD treatment significantly increased the expression of MMP9, MAPK3, PTGS2, JUN, IL1B and TNF compared with the control group (Figure 11B, C and D). Instructively, pretreatment of the cells with 10 μM luteolin sharply decreased the levels of MMP9, MAPK3, PTGS2, JUN, IL1B and TNF, suggesting that luteolin protects from OGD-induced damage through decreasing the expression levels of these targets.

## 4. Discussion

Despite years of intensive research, effective treatment options for stroke are still limited. Progress has been made in reducing the rates of acute deaths but nevertheless, the incidence of serious long-term disability resulting from stroke places an enormous strain on family members and society in general. Therefore, searching for effective treatments that reduce the morbidity and mortality of stroke remains a pressing issue. As a promising and cutting-edge approach to drug discovery, network pharmacology has been developed to predict new drugs or disease-specific targets [43,44]. Indeed, this approach in complement with powerful downstream analyses has been widely adopted to promote the understanding of herbal medicines. Regarding the latter, molecular docking simulations provide a streamlined route for computer-aided new drug design by predicting the affinity binding modes through ligand-receptor interactions. This can accelerate the design and screening of drugs by providing a solid foundation for experimental-based testing [45]. Therefore, mechanistic studies of luteolin-against-stroke treatment, based on network pharmacology and molecular docking to simulate ligand-target binding, could provide a basis for the development of targeted drugs for stroke.

Based on PPI network analysis of stroke targets, we obtained 10 targets with the highest degree values which included TP53, TNF, CASP3, STAT3, MAPK3, CXCL3, FN1, IL6, VEGFA and ALB that have been established to be involved in the development of stroke. Thereafter we selected the top three clusters from the PPI network via the MCODE plugin and performed GO functional enrichment and KEGG pathway analysis. The genes derived from GO analysis showed enrichment in relevant pathways including cytokine-mediated signaling pathway, inflammatory response, positive regulation of gene expression, aging, response to hypoxia, positive proliferation response to drug, and positive regulation of cell. Moreover, both the TNF signaling pathway and the cytokine-cytokine receptor interaction were the two highly ranked pathways associated with stroke based on the results of KEGG analysis, indicating these pathways as crucial targets for the treatment of stroke. In addition, the 10 core targets mentioned above were exactly covered by the TNF signaling pathway and the cytokine-cytokine receptor interaction pathways, which verified the correctness of our analysis.

The next step involved determining the intersection between the 275 putative genes targeted by luteolin with the 1200 genes related to stroke. Based on the 95 crossover genes identified, PPI network analysis uncovered six core genes including MMP9, MAPK3, PTGS2, JUN, IL1B and TNF likely linking the activity of luteolin to effective stroke control. Moreover, according to GO analysis, the candidate target genes were found to be associated with energy pathways, metabolism and immune responses. Furthermore, based on KEGG analysis, the therapeutic effects of luteolin on stroke may have through various pathways like the TNF signaling pathway, which overlapped with the results of KEGG analysis of stroke targets. Finally, we verified the binding ability of the six core genes of stroke and luteolin through molecular docking simulation, with the results indicating stable ligand-protein interactions. Moreover, in the case of the key targets MMP9 and MAPK3, the results in vitro model using PC-12 cells showed that luteolin treatment could alleviate their induction following OGD treatment.

According to the above analysis, the potential therapeutic mechanism of luteolin in treating stroke with luteolin could be summarized as the following four major points.

### 4.1 Luteolin reduces neuroinflammatory responses

The cytokines IL-1β and TNF-α play an important role in regulating immune responses after ischemic stroke and are stroke potential therapeutic targets [46]. Microglia, as the resident immune cells in CNS, can be activated after stroke and initiate neuroinflammation by producing cytotoxic and inflammatory factors including IL-1β and TNF-α to thereby exacerbate brain damage [27]. Moreover, TNF-α increases inflammation in the brain microenvironment by attracting leukocytes through chemotaxis and causes other cell subtypes to produce adhesion molecules [47]. TNF-α can also synergistically increases the toxic impact of IL-1β[47].

The MAPK signaling pathway involves can be grouped into three main families of intracellular serine/threonine protein kinases that include extracellular signal-regulated kinase (ERK), p38, and c-Jun NH2-terminal kinase (JNK) [48]. MAPK signaling is integrally involved in cell responses to stress conditions and mediates numerous cellular processes affecting cell fate including cell death, apoptosis, differentiation, proliferation, cell growth, immune res ponses, and inflammatory reactions [49–51]. Notably, OGD-induced hippocampal neuronal damage is reduced by downregulating MAPK signaling induced via the drug oxysophocarpine by attenuating the expression of inflammatory factors [52]. In addition, MAPK signaling pathway downregulation was found to reduce LPS-induced pro-inflammatory responses, which in turn reduced microglial-mediated neuronal damage [53,54]. JNK is a key member of the MAPKs family, which is implicated in the inflammatory process [55]. Moreover, inhibition of Erk2 reduced brain infarction, neuronal damage and inhibited oxidative stress, inflammatory response in the middle cerebral artery occlusion (MCAO) stroke mouse model [52].

PTGS2, also called cyclooxygenase (COX) 2, is the key rate-limiting enzyme that converts arachidonic acid into prostaglandins [56,57], such as prostaglandin E2 (PGE2), which acts as an efficient pro-inflammatory mediator [58]. Several studies have now associated PTGS2 expression and activity with ischemic stroke, where its high expression promotes inflammation [59–61]. Furthermore, miRNA-22 has been shown to reduce inflammatory damage caused by ischemic stroke by inhibiting the p38 MAPK/NF-κB pathway, thereby suppressing the expression of inflammatory factors such as PGE2, COX-2 and iNOS [62].

### 4.2 Luteolin reduces endothelial cell damage and BBB breakdown

MMPs are members of the zinc-dependent proteolytic enzymes family and are essential for the process of blood vessel formation as well as vascular remodeling [63]. However, where abnormal angiogenesis and vascular remodeling occur, excessive expression and activation of MMPs results in inflammatory responses [64]. Of the MMP family, MMP9, has been confirmed to be the major MMP member altered after ischemic stroke [65,66]. Expression of MMP-9 levels is high in brain tissue after stroke, and during the acute stage, high circulating MMP-9 levels are related to an increased risk of mortality, indicating a contribution of MMP-9 to ischemic brain injury [67,68]. Moreover, barrier permeability alterations are associated with increased expression of MMP-9 [65,66]. For example. Yang et al. observed the levels of MMP-9 mRNA and activity increased after reperfusion in MCAO-model rats, accompanied by BBB leakage and reductions in tight junctions in the cerebral cortex. Notably, inhibition of MMPs protects against the loss of tight junctions, proposing MMPs directly interfere with BBB integrity by degrading tight junction proteins. Furthermore, interleukins can also enhance the expression of MMPs, resulting in increased vascular permeability [69].

Additionally, TNF-α secreted from M1 phenotype microglia can cause endothelial necroptosis after MCAO, which also promotes vascular permeability and BBB breakdown [70]. ILs can also enhance the expression of MMPs, resulting in increased vascular permeability [71,72]. Nimesulide, the cyclooxygenase-2 inhibitor, can limit BBB destruction after cerebral ischemia by inhibiting COX-2 [73]. In addition, the COX-2 inhibitor CAY10404 reduces BBB injury after ischemia damage by decreasing MMP-9 activity and neutrophil infiltration [74]. Lastly, JNK has also been reported to be related to BBB breakdown [75].

### 4.3 Luteolin reduces apoptosis and oxidative stress

Oxidative stress decreases neuronal cell death by associating it with activation of the intracellular JNK signaling pathway [76]. Stress from ischemia increases sodium-glucose transporter type 1 (SGLT-1) in the brain, and SP600125 (JNK inhibitor) ameliorated ischemic neuronal damage and significantly inhibited the increase in SGLT-1 12 hours after MCAO [77]. Edaravone was used to treat acute ischemic stroke as a potent antioxidant, which has been reported to inhibit oxidative stress and the JNK-c-Jun pathway and simultaneously inhibit overall MAPK activity in the aged rat brain, which can greatly reduce neuronal damage after I/R injury [78].

The expression of COX-2 has been reported to increase sharply in the brain of ischemic rats from 30 minutes onwards and persisted until 15 days after stroke [74,79]. In addition, downregulation of the MAPK signaling pathway reduces cerebral ischemia/reperfusion injury and helps inhibit neuronal apoptosis [80].

### 4.4 Luteolin reduces the formation of thrombosis and infarct volume

It is known that the sensitivity of COX-2 knockout (COX-2-/-) mice to ischemic brain injury is sharply reduced [81]. Conversely, COX-2 overexpression increases infarct volume, which has been found to be associated with a sharp increase in PGE2 levels in the ischemic brain [82]. Lowering COX-2 expression is reported to be important in the treatment of stroke by decreasing the extent of ischemic brain injury after cerebral infarct [83]. In addition, TNF-α significantly enhances thrombosis by increasing the expression levels of plasminogen-activating inhibitor-1 tissue factor and platelet-activating factor [47].

Ours results demonstrated that luteolin can decrease the expression of MMP9, MAPK3, PTGS2, JUN, IL1B and TNF to maintain neuronal cell viability following an ischemic insult. We related the neuroprotective effects of luteolin to reductions in neuroinflammatory responses, endothelial cell damage, BBB breakdown, apoptosis, oxidative stress, thrombus formation and infarct volume. Together our findings propose the utility of luteolin as a therapeutic drug for ischemic stroke. And an important point is that, compared with other studies, our study abandoned the classical onset and injury mechanism to explore the possible mechanism of luteolin against ischemic stroke. Instead, we directly captured the rich representation of stroke-related targets from extensively annotated databases and combined these with the targets of luteolin. The intersection of these data enables candidate target selection in a less biased and visual way. Streamlining the understanding of the neuroprotective effects of luteolin on ischemic stroke provides several key benefits, not only reducing the time, financial and human costs required for trials and errors, but also providing new therapeutic ideas that undoubtedly will be of great value for the treatment of ischemic stroke.

## 5. Conclusion

From a systematic perspective, our study explored the molecular and pharmacological mechanisms of luteolin as a therapeutic agent to promote stroke recovery. However, there are still several limitations to network pharmacology and the companion methods used in our study. Ischemic stroke is a multifaceted process and in vitro experiments such as those used here are unlikely to fully capture this complexity. Furthermore, the research of new effective treatment opinions should not be limited in neuroprotection, but rather also for related processes including neuroregeneration, enhancement of repair and recovery, and maintenance of long-term neuroplasticity. Consequently, additional in vivo and clinical studies experiments are needed to verify the mechanisms disclosed by our research. Nonetheless, our study provides new directions for stroke patient recovery through promoting innovative research involving anti-stroke drugs.

## Figures and Tables

**Table:** 

TCMSP	Traditional Chinese Medicine Systems Pharmacology Database and Analysis Platform
GO	Gene Ontology
KEGG	Kyoto Encyclopedia of Genes and Genomes
PPI	protein-protein interaction
OB	oral bioavailability
DL	drug-likeness
BBB	blood-brain-barrier
TTD	Therapeutic Target Database
MCC	maximal clique centrality
MNC	maximum neighborhood component
BP	biological processe
CC	cellular component
MF	cellular component
DMEM	Dulbecco’s Modified Eagle Medium
CNS	Canadian Neurological Scale
MMSE	Mini-Mental State Examination
qRT-PCR	Quantitative reverse-transcription polymerase chain reaction
DAVID	Database for Annotation, Visualization and Integrated Discovery
IL-1β	interleukins-1β
TNF	tumor necrosis factor
MAPK	Mitogen-activated protein kinase
ERK	extracellular signal-regulated kinase
JNK	c-Jun NH2-terminal kinase
MMP	Matrix metalloproteinase
PTGS2	Prostaglandin H synthase 2
COX	cyclooxygenase
PGE2	prostaglandin E2
MCAO	middle cerebral artery occlusion
SGLT-1	sodium-glucose transporter type 1
OGD	oxygen and glucose deprivation

## Research highlights

1. Luteolin targets MMP9, MAPK3, PTGS2, JUN, IL1B and TNF to downregulate their expression.

2. Luteolin produces neuroprotective effects by supporting neuron viability after OGD.

3.Understanding targets and mechanisms provide new directions for stroke treatment.
